# Examining antecedents of organizational citizenship behavior: An empirical study in Indonesian police context

**DOI:** 10.1371/journal.pone.0291815

**Published:** 2023-10-05

**Authors:** Ahmad Rizki Sridadi, Anis Eliyana, Fika Arista Priyandini, Andika Setia Pratama, Shochrul Rohmatul Ajija, Nurul Liyana Mohd Kamil

**Affiliations:** 1 Department of Management, Universitas Airlangga, Surabaya, East Java, Indonesia; 2 Department of Economics Science, Universitas Airlangga, Surabaya, East Java, Indonesia; 3 Department of Public Administration, Universiti Malaya, Kuala Lumpur, Malaysia; National University of Sciences and Technology, PAKISTAN

## Abstract

Police reform in the Mobile Brigade Corps unit in Indonesia, which seeks to break away from militaristic elements, has not been fully implemented optimally. This is reflected in the lack of implementation of human values in serving the community. The extra effort of officers in encouraging community service based on human values can be realized through Organizational Citizenship Behavior which is not only directed at fellow officers, but also towards organizations in the context of community service. Based on Social Exchange Theory, this study aims to investigate the mechanism of strengthening Organizational Citizenship Behavior in the context of the Police Mobile Brigade Corps with the support of Empowering Leadership, Psychological Empowerment, and Job Satisfaction. Using a quantitative approach, this study distributed online questionnaires to 395 Mobile Brigade Corps officers. Furthermore, this study analyzes the data using Partial Least Squares–Structural Equation Modeling. The test results show that Empowering Leadership can strengthen Organizational Citizenship Behavior. In addition, this study reveals the mediating role of Psychological Empowerment and Job Satisfaction in the influence of Empowering Leadership on Organizational Citizenship Behavior. With these findings, the Police Mobile Brigade Corps needs to improve the competence of officers through training and development efforts so that officers feel psychologically empowered and have job satisfaction. On the other hand, Mobile Brigade Corps needs to encourage leaders to provide opportunities for officers to participate in decision making and recognize their contributions to work.

## Introduction

Since being legalized in 2002 as a separate entity from the army force, the police in Indonesia have been committed to carrying out organizational reforms. It was done to achieve proportionality, professionalism, and complete police integrity. This reform also marked the need for a change in police culture to prioritize service and interpersonal relations with the community. However, reform in the police has yet to take place in its entirety. As part of the organization’s history, the military element is still attached to the policing culture. This also applies to a special unit within the Indonesian police tasked with dealing with high-risk security threats, namely the Police Mobile Brigade Corps. Unfortunately, this unit has not made any real improvements in public services in Indonesia [1]. The Police Mobile Brigade Corps also has yet to show any change concerning police force reform [2]. In addition, there are still limitations in the ability and expertise of this force to build interpersonal relationships with the community. It reflects the low implementation of the service motto, “*Jiwa ragaku untuk kemanusiaan*” ("My Body and Spirit for Humanity"). Therefore, the implementation of service values in order to support reform within Police Mobile Brigade Corps needs to be improved.

In order to achieve this, Police Mobile Brigade Corps units can instill self-sacrificing behavior and voluntarily help other co-workers, the community, and the state. This behavior also includes actions beyond their main duties without expecting anything in return, known as Organizational Citizenship Behavior. This behavior is defined as voluntary individual behavior, has no formal compensation system, and is carried out to support the effectiveness of organizational functionality [3]. Organizational Citizenship Behavior is proven to provide many benefits, both for other individuals and the organization itself [4]. The Police Mobile Brigade Corps needs organizational Citizenship Behavior to increase strong bonds with other officers and the organization. There are several factors that can encourage Organizational Citizenship Behavior: the individual, the job, the leader, and the organization [4].

Furthermore, the increase in Organizational Citizenship Behavior is influenced by two main factors, namely internal factors, which include job satisfaction, positive attitudes, and psychological acceptance (Psychological Empowerment), as well as external factors, such as Empowering Leadership and management systems [5]. These factors correlate to Social Exchange Theory (SET). SET emphasizes a series of interactions that give rise to "obligations" [6]. The interaction can be the relationship between the leaders and followers. A certain treatment from the leader towards the followers will be reciprocated in the same form by the followers [7]. In this case, Empowering Leadership is expected to encourage job satisfaction and psychological empowerment of officers that lead to Organizational Citizenship Behavior Empowering leadership emphasizes how leaders influence subordinates through sharing power, motivational support, and development to promote experiences of independence, motivation, and the ability to work independently, limited to overall organizational goals and strategies [8]. Furthermore, Empowering Leadership aims to reduce militaristic behavior among officers and create extra-role behavior that can foster effectiveness in this force. Referring to SET, extra-role is a reciprocity for the treatment of leaders who empower officers. The form of Empowering Leadership is the commander who always helps Police Mobile Brigade Corps officers understand their role, their duties, and the alignment of unit goals at every morning assembly.

Besides Empowering leadership, other factors play a role in shaping Organizational Citizenship Behavior, namely Psychological Empowerment. Psychological empowerment is defined as a condition that describes the extent to which individuals feel that they have competence related to the work, control the decisions taken in work, and obtain the impact and meaning of the work [9]. High self-confidence in carrying out their duties in handling riots, social conflicts, and demonstrations can inspire these officers to help other colleagues and units voluntarily with confidence in their adequate competence to carry out tasks beyond this obligation. It is supported by Psychological Empowerment, which has a strong positive influence on Organizational Citizenship Behavior [10].

In addition to the role of the Empowering Leadership and Psychological Empowerment variables, the role of Job Satisfaction also influences Organizational Citizenship Behavior. Job Satisfaction is an emotional state leading to a feeling of pleasure due to the work achievement that has been done and assessed [11]. Job Satisfaction can be interpreted as a form of individual perception of how well their job provides important things [12]. With this satisfaction, Police Mobile Brigade Corps officers are motivated to do things that are beneficial to the unit in return and, in turn, can realize real reforms. It is supported by changes in facilities and infrastructure, rewards, punishments, motivation, and training [13]. Job Satisfaction also directly impacts individual OCB and Organizational OCB [14].

Concerning the role of mediation, several studies have found that Psychological Empowerment plays a role in mediating the effect of Empowering Leadership on Organizational Citizenship Behavior. Psychological empowerment is proven to fully mediate the influence of Empowering Leadership and Organizational Citizenship Behavior [15]. Then, Psychological Empowerment shows partial mediation in the influence between Empowering Leadership and Organizational Citizenship Behavior on several subordinates in seven major European organizations [16]. On the other hand, there is no mediating role of Psychological Empowerment on the effect of Empowering Leadership and Organizational Citizenship Behavior in the context of organizational employees in the telecommunications sector [17] and the hotel property sector [18].

Meanwhile, Job Satisfaction also has a mediating role that influences Empowering Leadership and Organizational Citizenship Behavior with some of the previous findings. Job Satisfaction is proven to have a fully mediating role in the influence between Empowering Leadership and Organizational Citizenship Behavior [19]. Then, Job Satisfaction is proven to partially mediate the effect of empowerment on Organizational Citizenship Behavior in service sector organizations [20]. Conversely, there are findings that Job Satisfaction does not mediate the effect of empowerment on Organizational Citizenship Behavior [21].

Based on various previous research findings, Psychological Empowerment and Job Satisfaction have inconsistencies in mediating the effect of Empowering Leadership on Organizational Citizenship Behavior in different organizational contexts. However, among the research that has been done, the context of the police is still not a concern. Therefore, this study aims to test and analyze Psychological Empowerment and Job Satisfaction on the effect of Empowering Leadership on Organizational Citizenship Behavior, specifically in Police Mobile Brigade Corps. Thus, it is important to conduct this research to find out how efforts to optimize Organizational Citizenship Behavior in the Police Mobile Brigade Corps can bridge the goals of realizing Police force reform.

## Literature review and hypothesis development

This section will present a conceptual explanation of Empowering Leadership, Psychological Empowerment, Job Satisfaction, and Organizational Citizenship Behavior. In addition, this section also explains the development of hypotheses based on the SET framework.

### Empowering leadership and Organizational Citizenship Behavior

Empowering leadership is defined as behavior in which the leader shares power with his subordinates and increases the level of intrinsic motivation of the subordinates [22]. Furthermore, Empowering Leadership is also defined as the behavior of leaders who empower directly or indirectly through individual psychology to motivate them to have the desire to pay for these benefits and increase motivation and trust in leaders [23]. In this case, the leader also delegates duties and powers over his work to subordinates in the organization. When subordinates are empowered by having great control at work, they can encourage them to feel a deeper meaning for what has been done in their work. Empowering leadership becomes an organizational stimulus to influence its members or team [24]. Thus, they can foster a high sense of self-confidence and competence to be loyal to the organization. In turn, subordinates are willing to voluntarily carry out tasks outside their obligations.

**Fig 1:**
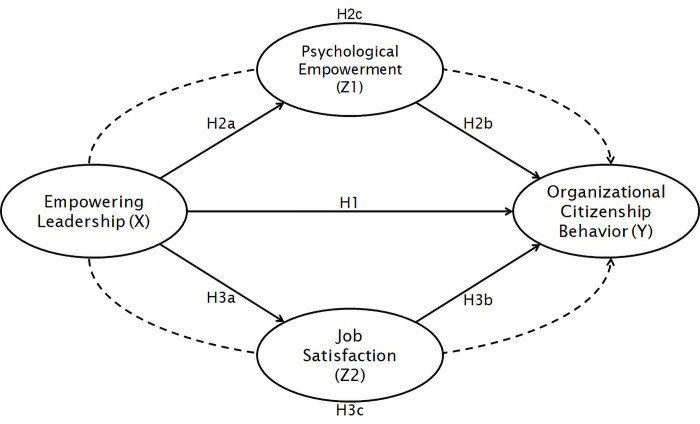
Conceptual framework.

Meanwhile, Organizational Citizenship Behavior is defined as individual behavior that has a discretionary nature, there is no formal compensation system, and this behavior is carried out to support the effectiveness of organizational functionality [3]. Organizational Citizenship Behavior is individual behavior that shows a willingness to be involved in an extra role in the organization [25]. In this case, empowered individuals can foster a high sense of self-confidence and competence so that they are willing to carry out tasks outside their obligations voluntarily without any compensation for the sake of the organization. Based on SET, the extra-role behavior becomes a form of good response from the individual to the treatment received from the empowering leader. It is supported by prior research which found that empowering leadership directly affects Organizational Citizenship Behavior [15] Empowering leadership is also considered capable of influencing Organizational Citizenship Behavior in the context of the manufacturing division’s subordinates [26]. Nonetheless, previous findings state that there is no direct influence between Empowering Leadership and Organizational Citizenship Behavior [27]. Thus, the first hypothesis of this study can be stated as follows (see Fig 1):

*H1*: *Empowering Leadership has a significant effect on Organizational Citizenship Behavior*.

### Empowering leadership, psychological empowerment, and Organizational Citizenship Behavior

Psychological empowerment is defined as a work motivation construct manifested in four cognitions: meaning, competence, self-determination, and impact [28]. In other words, Psychological Empowerment is a psychological state of an individual who feels that there are four dimensions of psychological empowerment, including meaningfulness, competence, self-determination, and impact, which are influenced by the behavior of empowering leaders in the organization [29]. In recent research, Psychological Empowerment is interpreted as a condition where there is self-efficacy in organizational members which is characterized by identifying and eliminating conditions that are considered unproductive [30]. Leaders with Empowering Leadership tend to increase the meaning of work for their subordinates to help them understand how important their contribution is to the organization, which can foster Psychological Empowerment. It is supported by previous findings, which state that Empowering Leadership is proven to have a strong positive influence on Psychological Empowerment [31]. Then, from the perspective of hospital nurses, Empowering Leadership also affects the increase in Psychological Empowerment [32].

Furthermore, individuals with high Psychological Empowerment feel that they are properly empowered in their workplace to foster individual motivation and confidence in helping other things outside of their main duties with their abilities. With this high psychological empowerment, individuals do not hesitate to do things outside their obligations. It is supported by previous findings that Psychological Empowerment positively influences Organizational Citizenship Behavior in the context of teachers [33]. Furthermore, Psychological Empowerment is considered to strongly influence Organizational Citizenship Behavior in the context of the manufacturing division’s subordinates [26].

With the direct influence of Empowering Leadership on Psychological Empowerment and Psychological Empowerment on Organizational Citizenship Behavior, it can be assumed that Psychological Empowerment is a variable that mediates Empowering Leadership on Organizational Citizenship Behavior. In accordance with SET, Psychological Empowerment that is formed as a result of Empowering Leadership will trigger awareness for individuals to contribute back to leaders and organizations in the form of Organizational Citizenship Behavior. Apart from the evidence of direct influence, previous findings have shown that Psychological Empowerment significantly mediates the effect of Empowering Leadership and Organizational Citizenship Behavior on the large organizational sector in China [34]. Thus, the second hypothesis in this study can be stated as follows (see Fig 1):

*H2a*: *Empowering Leadership has a significant effect on Psychological Empowerment*.
*H2b*: *Psychological empowerment has a significant effect on Organizational Citizenship Behavior*.
*H2c*: *Psychological empowerment significantly mediates the effect of Empowering Leadership on Organizational Citizenship Behavior*.

### Empowering leadership, job satisfaction, and Organizational Citizenship Behavior

Job Satisfaction is defined as an individual’s emotional state that leads to pleasure as a result of an assessment of the work done and the values achieved in the job [11]. In this case, Job Satisfaction is felt by individuals when they feel that the work has been successful. Conversely, Job Dissatisfaction occurs when individuals feel disappointed with the work done and do not achieve any value. Job Satisfaction is also an emotional and cognitive state toward assessing work’s internal and external aspects [35]. Therefore, highly Empowering Leadership leaders can empower individuals so that they feel satisfied with what is done in their work. High Empowering Leadership will cause Job Satisfaction [36].

As for Job Satisfaction, it is proven to be able to provide benefits for both individuals and organizations, including being able to encourage Organizational Citizenship Behavior. In this case, individuals with high levels of job satisfaction can encourage to cultivate the behavior of wanting to work extra beyond what is assigned to them to help the smooth running of the organization. It is supported by previous findings, which state that Job Satisfaction has a positive effect on individual OCB and Organizational OCB [14, 37].

With the direct influence of Empowering Leadership on Job Satisfaction and Job Satisfaction on Organizational Citizenship Behavior, it can be assumed that job satisfaction is a variable that mediates Empowering Leadership on Organizational Citizenship Behavior. Referring to SET, individuals are happy to do Organizational Citizenship Behavior as a reward for feeling satisfied in their work that is created because of Empowering Leadership. Apart from the fact that there is evidence of a direct effect, there is research that states significant results where Job Satisfaction has a mediating role in the influence between Empowering Leadership and Organizational Citizenship Behavior [38]. Thus, the third hypothesis in this study can be stated as follows (see Fig 1):

*H3a*: *Empowering Leadership has a significant effect on Job Satisfaction*.
*H3b*: *Job Satisfaction has a significant effect on Organizational Citizenship Behavior*.
*H3c*: *Job Satisfaction significantly mediates the effect of Empowering Leadership on Organizational Citizenship Behavior*.

## Research methods

This section describes the research method consisting of the mechanism of data collection and analysis.

### Data collections

Data collection in this study was carried out through a survey by distributing questionnaires online (Google Form) to all Police Mobile Brigade officers in Indonesia. The sampling technique in this study was simple random sampling. Simple random sampling is a sampling technique which gives equal opportunity to all members of the population to be sampled. This technique was chosen because it is capable of producing high generalization of research results compared to other sampling techniques [39]. The survey results found 395 Police Mobile Brigade Corps officers who filled out the complete questionnaire. This number meets the minimum sample criteria of 10 times the number of indicators [40]. In this study, there are 38 indicators to measure research variables so that the minimum number of samples according to these criteria is 380 respondents.

The Research Ethics Committee at Universitas Airlangga: Development and Innovation Institute for Publishing Journal and Intellectual Property Rights (LIPJIPHKI) has confirmed that no ethical approval is necessary for research that includes no treatment and involves non-vulnerable participants. In accordance with the organization’s policy, the Chief has granted written informed permission on behalf of all respondents. All respondents were notified before completing the questionnaires that data collection was voluntarily. Furthermore, they agreed upon the written consent given prior to the survey. In addition, respondents were notified that their replies would be absolutely confidential and utilized for research purposes only. LIPJIPHKI has also validated the consent.

### Measurement

In its measurement, this study uses a 5-point Likert measurement scale to measure each variable measurement indicator. Empowering leadership is measured using nine indicators with three dimensions consisting of Enhancing Meaningfulness of Work (EMW) such as "The Police Mobile Brigade Corps commander helps me understand how the relationship between the officer’s and the Police Mobile Brigade Corps’s goals"; Expressing Confidence in High Performance (ECHP) such as "When I make a mistake, the commander believes that I can be better with the skills I have"; and Providing Autonomy from Bureaucratic Constraints (PABC) such as "The commander makes work activities more efficient by making simple rules” [31]. Furthermore, Psychological Empowerment is measured using 12 indicators with four dimensions consisting of *Meaning* such as "For me, the work I do is very important", *competence* such as "I am confident in my ability to work", *Self-determination* such as "I have autonomy in determining how to do my job”, and *impact* like “I have a contribution to influence what happens in Police Mobile Brigade Corps " [28]. Then, Job Satisfaction is measured using ten indicators, such as "I receive recognition for a well-completed job" [41]. Finally, Organizational Citizenship Behavior is measured using seven indicators with two dimensions consisting of OCB-Individual, such as "I help colleagues who have more workload," and OCB-Organization, such as "I carry out informal rules when working" [10, 42]. All measurement indicators were then translated into Indonesian and were content validated first by a police expert in Indonesia.

### Data analysis technique

This study uses the Partial Least Square–Structural Equation Model (PLS-SEM) analysis technique with the second-order level and is supported by SmartPLS 3 software. PLS-SEM is a variance-based statistical SEM method that can perform measurement and structural model testing simultaneously [40]. This study uses PLS-SEM because it is capable of accurately estimating complex models with a large sample size. In addition, PLS-SEM is able to test constructs consisting of several indicators. In guaranteeing robustness, PLS-SEM provides various criteria to ensure the accuracy of model test results.

Furthermore, there are two analysis models in PLS-SEM, namely outer (measurement) model, and inner (structural) model. The outer model is used to ensure the validity and reliability of measurement instruments. Instrument validity was measured by two criteria, namely convergent validity and discriminant validity. Convergent validity indicates the extent to which each indicator is able to measure the variable that should be measured. Meanwhile, discriminant validity shows the difference of a construct compared to other constructs in one model. Furthermore, reliability testing is carried out to ensure the reliability of the measuring instrument. Meanwhile, the inner model analyzes the direction of influence between the variables. Furthermore, the analysis was carried out using the second-order level, which involved examining a high-level structure containing two layers of construction [40].

## Results

This section describes the research findings which include a description of the demographics of the respondents, the results of testing the outer model, and the inner model.

### Respondent description

A total of 395 respondents have completely filled out the research questionnaire. The demographic description of the respondents in this study is in Table 1.

**Table 1:** Respondent demography.

Characteristics	Category	N	Percent
Gender	Male	378	95.7%
	Female	17	4.3%
Age	20–30 years	213	53.9%
	31–40 years	78	19.7%
	41–50 years	96	24.3%
	> 50 years	8	2.0%
Tenure	< 1 year	159	40.3%
	1–3 years	35	8.9%
	4–6 years	12	3.0%
	7–9 years	32	8.1%
	10–12 years	53	13.4%
	13–15 years	52	13.2%
	> 15 years	52	13.2%
Highest Education	High School	315	79.7%
	Diploma	6	1.5%
	Bachelor	70	17.7%
	Master	4	1.0%

Based on Table 1, the respondents in this study were dominated by men, amounting to 95.7%. In addition, around 53.9% of all respondents are aged 20–30 years. The majority of respondents (40.3%) tend to have worked as Police Mobile Brigade Corps officers for less than one year. With regard to last education, the majority of respondents were high school graduates, amounting to 79.7%.

### Outer model testing

This study tested the outer model to determine the validity and reliability of the measurement indicators used in measuring each variable. The validity test refers to convergent validity and discriminant validity. Meanwhile, reliability testing refers to internal consistency reliability.

### Convergent validity

Convergent validity is measured based on the outer loading value of each measurement indicator which must be > 0.70, and the Average Variance Extracted (AVE) must be > 0.50 [40]. The results of the convergent validity tests are shown in the following table.

Table 2 shows that all measurement indicators for each variable have outer loading values > 0.70 and AVE > 0.50, meaning that the measurement indicators in this study have been able to explain or measure variables appropriately. Therefore, all variable measurements have met convergent validity.

**Table 2:** Convergent validity.

Variables	Dimensions	Indicators	Validity Test			
			Outer Loading	Result	AVE	Result
Empowering Leadership (X)	EMW	EMW1	0.862	Valid	0.717	Valid
		EMW2	0.908	Valid		
		EMW3	0.880	Valid		
	ECHP	ECHP1	0.846	Valid		
		ECHP2	0.766	Valid		
		ECHP3	0.923	Valid		
	PABC	PABC1	0.859	Valid		
		PABC2	0.793	Valid		
		PABC3	0.766	Valid		
Psychological Empowerment (Z1)	Meaning	M1	0.773	Valid	0.651	Valid
		M2	0.816	Valid		
		M3	0.814	Valid		
	Competence	C1	0.829	Valid		
		C2	0.813	Valid		
		C3	0.813	Valid		
	Self-determination	SD1	0.769	Valid		
		SD2	0.873	Valid		
		SD3	0.785	Valid		
	Impact	I1	0.800	Valid		
		I2	0.818	Valid		
		I3	0.752	Valid		
Job Satisfaction (Z2)		JS1	0.815	Valid	0.714	Valid
		JS2	0.835	Valid		
		JS3	0.833	Valid		
		JS4	0.873	Valid		
		JS5	0.886	Valid		
		JS6	0.839	Valid		
		JS7	0.916	Valid		
		JS8	0.824	Valid		
		JS9	0.799	Valid		
		JS10	0.821	Valid		
Organizational Citizenship Behavior (Y)	OCB- Individual	OCBI1	0.890	Valid	0.670	Valid
		OCBI2	0.808	Valid		
		OCBI3	0.805	Valid		
		OCBI4	0.789	Valid		
	OCB-Organizational	OCBO1	0.819	Valid		
		OCBO2	0.807	Valid		
		OCBO3	0.807	Valid		

### Discriminant validity

Discriminant validity testing is based on the Heterotrait-Monotrait Ratio (HTMT) [43]. The HTMT value <0.90 indicates that a construct has no similarities in one model. The results of discriminant validity testing with HTMT in this study can be seen in Table 3.

**Table 3:** Discriminant validity.

	Empowering Leadership (X)	Psychological Empowerment (Z1)	Job Satisfaction (Z2)
Psychological Empowerment (Z1)	0.737	-	-
Job Satisfaction (Z2)	0.709	0.875	-
Organizational Citizenship Behavior (Y)	0.752	0.871	0.843

Based on Table 3, it can be seen that the HTMT value for each variable is less than 0.90. It shows that the variables in this study are not similar in terms of the measurement instruments. Therefore, the measurement instrument meets discriminant validity.

### Internal consistency reliability

Furthermore, the reliability test is guided by the value of Cronbach’s alpha and composite reliability. The instrument is declared reliable if it has a Cronbach’s alpha value and composite reliability > 0.60 [40]. The results of testing the reliability of the instrument in this study are in Table 4.

**Table 4:** Reliability test.

Variables	Cronbach’s alpha	Composite Reliability
Empowering Leadership (X)	0.913	0.958
Psychological Empowerment (Z1)	0.910	0.957
Job Satisfaction (Z2)	0.932	0.961
Organizational Citizenship Behavior (Y)	0.893	0.934

The results of the instrument reliability test in Table 4 show the value of Cronbach’s alpha and composite reliability is > 0.60. It can be interpreted that the research instrument has good consistency in measuring the variables studied. Thus, the measurement results can be the basis for carrying out further tests.

### Inner model testing

In this study, four types of inner model tests need to be carried out, which consist of the coefficient of determination (R^2^), effect size (f^2^), cross-validated redundancy (Q^2^) and hypothesis test. The Coefficient of Determination is carried out in research to know how accurately the independent variables can influence the dependent variable. Furthermore, Effect Size is carried out in research to know changes in the value of R^2^ if an independent variable is removed from the model. Then, Cross-validated Redundancy is carried out in research to determine whether there is predictive relevance in the dependent variable. The test results for the coefficient of determination (R^2^), effect size (f^2^), and cross-validated redundancy (Q^2^) are shown in the following table.

Table 5 shows that the variable has a value of R^2 in the medium to high category with values > 0.50 and > 0.75, illustrating the high accuracy of the effect of the independent variable on the dependent variable. Meanwhile, the f^2 value indicates that the effect of Empowering Leadership has the smallest contribution directly to changes in Organizational Citizenship Behavior values, and Job Satisfaction has the largest direct contribution to changes in Organizational Citizenship Behavior values. Then, the value of Q^2 fully meets the criteria with a value of 0, so it has great predictive relevance.

**Table 5:** Inner model test.

Variables	*R* ^*2*^	*f* ^*2*^	*Q* ^*2*^
Empowering Leadership (X)		0.054	
Psychological Empowerment (Z1)	0.708	0.155	0.223
Job Satisfaction (Z2)	0.629	1.030	0.264
Organizational Citizenship Behavior (Y)	0.881		0.418

### Hypothesis testing

**Fig 2:**
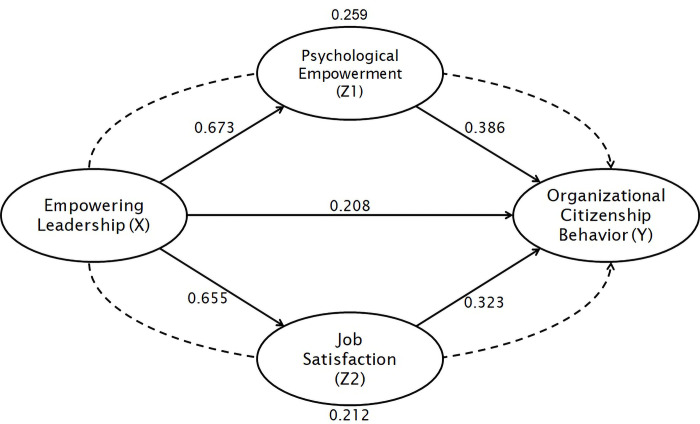
Path diagram.

Hypothesis testing is carried out by bootstrapping to determine the magnitude of the coefficient of influence of Empowering Leadership on Organizational Citizenship Behavior both directly and indirectly through Psychological Empowerment and Job Satisfaction. The effect between variables is said to be significant when it has a coefficient value > 0, P-value < 0.05, and t statistic > 1.96 [40]. The results of testing the research hypothesis are presented in Table 6 and Fig 2:

**Table 6:** Hypothesis testing results.

Effect(s)		Coefficient	*t-statistic*	*P-value*	Results
H1	Empowering Leadership (X) → Organizational Citizenship Behavior (Y)	0.208	3.351	0.000	Significant
H2a	Empowering Leadership (X) → Psychological Empowerment (Z1)	0.673	16.265	0.000	Significant
H3a	Empowering Leadership (X) → Job Satisfaction (Z2)	0.655	15.485	0.000	Significant
H2b	Psychological Empowerment (Z1) → Organizational Citizenship Behavior (Y)	0.386	5.750	0.000	Significant
H3b	Job Satisfaction (Z2) → Organizational Citizenship Behavior (Y)	0.323	4.707	0.000	Significant
H2c	Empowering Leadership (X) → Psychological Empowerment (Z1) → Organizational Citizenship Behavior (Y)	0.259	5.346	0.000	Partial Mediation
H3c	Empowering Leadership (X) → Job Satisfaction (Z2) → Organizational Citizenship Behavior (Y)	0.212	4.474	0.000	Partial mediation

Based on Table 6, it can be seen that the test results show that the direct effect of Empowering Leadership on Psychological Empowerment, Job Satisfaction, and Organizational Citizenship Behavior is proven to be significant with a t-statistic > 1.96 and P-value (0.000) < 0.05 meaning that H1, H2a, and H3a are accepted. Furthermore, the direct effect of Psychological Empowerment and Job Satisfaction on Organizational Citizenship Behavior is significant, with a t-statistic > 1.96 and P-value (0.000) < 0.05, so H2b and H3b are accepted. Thus, the mediating effect produced by Psychological Empowerment and Job Satisfaction is partial mediation.

## Discussion

The results of this study prove that there is a direct influence between Empowering Leadership on Organizational Citizenship Behavior. Previous research has shown that empowering leadership can encourage organizational citizenship behavior [15]. In this context, commanders empower officers by encouraging officer participation in decision-making thereby reducing bureaucratic tightness. Bureaucratic leniency can also create positive behavior in the form of the willingness of officers to voluntarily perform extra-role for the sake of troop effectiveness. Furthermore, the application of Empowering Leadership is manifested in dimensions like EMW, ECHP, and PABC. These three dimensions emphasize increasing the meaningfulness of work, recognizing the competence of the Police Mobile Brigade officers, and simplifying the bureaucracy. This was seen by the officers as good treatment by the leadership towards him. Compliant with SET, these forms of Empowering Leadership can build the extra-role behavior of officers both towards individuals and organizations. Thus, the results of this study support previous findings, which prove that Empowering Leadership has a significant effect on Organizational Citizenship Behavior [26, 44]. Empowering Leadership is able to have a positive effect on Organizational Citizenship Behavior on individuals in teams [34].

Not only influence Organizational Citizenship Behavior directly, but Empowering Leadership also has a direct effect on Psychological Empowerment. Leaders that encourage participation in decision-making and provide bureaucratic leniency can make officers experience meaningful work, recognition of self-competence, determination, and control in decision-making, as well as unity as a form of psychological empowerment they receive. Previous research has proven that empowering leadership contributes to increasing psychological empowerment in the context of hospital nurses [32]. These results are consistent with this study. As for the highly Empowering Leadership and Psychological Empowerment of officers are represented by the dimensions that make up the two variables. Empowering leadership is realized in three dimensions: EMW, ECHP, and PABC. The existence of meaningful work, competence in work, and job autonomy can impact psychological conditions due to the empowerment received in the form of Meaning, Competence, Self-determination, and Impact. Thus, the results of this study support previous findings which prove that Empowering Leadership has a significant effect on Psychological Empowerment [16, 31, 45]. The results of this study are also in line with the statement that empowering leadership can provide great benefits in the public sector in the form of reducing individual dependence on superiors for sustainable decision making, work direction, emotional aspects of leadership, and the loss of subordinate innovation barriers [46].

Furthermore, Empowering Leadership has also been shown to influence Job Satisfaction directly. In other words, Empowering Leadership is proven to make officers feel high Job Satisfaction. Previous research stated that empowering leadership has a positive effect on job satisfaction from the perspective of subordinates and administrators in higher education institutions [36]. These results are consistent with the results of this study in the context of the Police Mobile Brigade Corps in Indonesia. Furthermore, a commander that focuses on empowerment by encouraging the participation of the officers in decision-making and bureaucratic leniency can create a positive emotional state as a response to the work evaluation that has been done. The high level of Empowering Leadership is realized through the three dimensions that make it up: EMW, ECHP, and PABC. With these things, Police Mobile Brigade Corps officers can feel empowered by the leaders, stimulating a sense of satisfaction with their work. Job satisfaction is reflected in their enjoyment of their co-workers and work. Thus, the results of this study support previous findings, which prove that Empowering Leadership has a significant effect on Job Satisfaction [19, 38, 47]. Thus, officers who feel a form of empowering leadership from a leader are able to experience high job satisfaction because they feel more trusted and valued.

Moreover, the results of this study found that Psychological Empowerment had a direct effect on Organizational Citizenship Behavior. It shows that the psychological empowerment received by the officers in the form of meaningful work, recognition of self-competence, determination, and control in decision-making, and the impact can encourage them to voluntarily act on their duties and responsibilities for the effectiveness of the organization. In line with the findings of previous studies, psychological empowerment has a strong influence on organizational citizenship behavior in the context of manufacturing division subordinates [26]. In addition, it is known that psychological empowerment is able to encourage the volunteering of subordinates to carry out extra-roles in their work or in other words organizational citizenship behavior [33]. Thus, the results of this study support previous findings, which proved that Psychological Empowerment proved to have a significant effect on Organizational Citizenship Behavior [10, 48].

Not only does Psychological Empowerment show its influence on Organizational Citizenship Behavior, but Job Satisfaction also shows an influence on Organizational Citizenship Behavior. In this context, the officers with a positive emotional state leading to a high degree of satisfaction can encourage the behavior to be willing to do things outside of their duties and functions for organizational effectiveness. Previous research stated that job satisfaction has a positive effect on Individual OCB and Organizational OCB based on the context of subordinates working in the public and private sectors [37]. This research also proves that job satisfaction which is represented by a sense of satisfaction with the job they have and good relations with co-workers, means that Police Mobile Brigade Corps officers do not hesitate to do something outside their duties and functions to help the effectiveness of troops as a form of feedback. Thus, the results of this study support previous findings, which prove that Job Satisfaction has a positive and significant effect on Organizational Citizenship Behavior [14, 49, 50]. This can happen because job satisfaction can influence individual performance for the better so that organizations can run more effectively [51], one of which is by increasing Organizational Citizenship Behavior [52].

The results of this study have provided evidence that Empowering Leadership directly affects Psychological Empowerment and Job Satisfaction. Both have proven to have a direct effect on Organizational Citizenship Behavior, so the mediating role of Psychological Empowerment and Job Satisfaction is full mediation in the influence between Empowering Leadership and Organizational Citizenship Behavior. Other research has corroborated the mediating role of psychological empowerment in the relationship between empowering leadership and organizational citizenship behavior in seven large European organizations [16]. In addition, it is known that job satisfaction mediates the relationship between the empowerment received by employees and supervisors and organizational citizenship behavior in service sector organizations [20].

Referring to SET, leaders who empower their subordinates will result in increased job satisfaction and psychological empowerment. In this situation, the leader instills confidence in followers by delegating tasks, thereby allowing followers to feel that the leader recognizes their competence. It results in adherents believing they are empowered by the leader and experiencing job satisfaction due to the leader’s support. In response to what they receive and how they feel, followers appear obligated to recompense Empowering Leaders with positive actions, such as Organizational Citizenship Behavior. In this case, Empowering Leadership can only sometimes make the officers want to do things beyond their main duties. Therefore, other things are needed to encourage this behavior, such as psychological conditions as a form of empowerment received and job satisfaction. Thus, the results of this study support several previous studies which have discussed the mediating role of Psychological Empowerment [15] and Job Satisfaction [19] in mediating the influence of Empowering Leadership and Organizational Citizenship Behavior.

## Implication

This study proved the direct influence of Empowering Leadership on Organizational Citizenship Behavior in the context of the Police Mobile Brigade Corps. The previous research, which discussed the direct effect of Empowering Leadership on Organizational Citizenship Behavior, was examined in the private sector.

In the context of this study, it is known that Empowering Leadership, Psychological Empowerment, and Job Satisfaction have contributed to encouraging Organizational Citizenship Behavior in the Pioneer Troops of the Police Mobile Brigade Corps. The commander with empowering leadership seeks to provide empowerment to Police Mobile Brigade Corps officers by encouraging participation, loosening the bureaucracy, and instilling a high sense of work, so that officers experience meaningful work, recognition of self-competence, determination, and control in decision-making, and the impact of Police Mobile Brigade Corps officers in unity as a form of psychological empowerment it receives. On the other hand, various forms of empowerment foster a sense of satisfaction for Police Mobile Brigade Corps officers with their work and those in the Police Mobile Brigade Corps. Under these conditions, the officers are encouraged to voluntarily do things outside of their main duties for the effectiveness of the Indonesian Police Mobile Brigade Corps. Therefore, Organizational Citizenship Behavior can support the Police Mobile Brigade Corps in realizing Police force reform and implementing service values.

Furthermore, the results of this study can be used as a reference in compiling activities and agendas related to concrete manifestations of reform within Police Mobile Brigade Corps, which can encourage the emergence of Organizational Citizenship Behavior supported by the influence of Empowering Leadership, Psychological Empowerment, and Job Satisfaction. Organizational Citizenship Behavior as a voluntary behavior is needed by the organization, especially the Police Mobile Brigade Corps, for its functional effectiveness. Organizational Citizenship Behavior is required by Police Mobile Brigade Corps, considering that the duties and functions are more challenging compared to the general police. This force deals with high-level and high-intensity disturbances, while the general police force focuses on maintaining public order and security. Therefore, the findings of this study can also be used by other countries with special forces that handle heavier tasks than the general police.

To encourage Organizational Citizenship Behavior, the Police Mobile Brigade Corps first prioritized Psychological Empowerment because it has proven to have the greatest contribution to Organizational Citizenship Behavior, with efforts to develop abilities, interests, talents, and skills. Furthermore, this force needs to focus on increasing Job Satisfaction through efforts to provide supporting facilities in the form of training and development to improve the capabilities and skills of Police Mobile Brigade Corps officers. In addition, Empowering Leadership has also proven to have positive benefits. Empowering leadership implemented by Police Mobile Brigade Corps commanders can assist them in carrying out their main tasks and functions by taking action on threats and security disturbances of high degree and intensity. Therefore, Empowering Leadership needs to be managed effectively and wisely by the organization.

## Conclusion

Based on the results of the tests and discussions described above, this study proves that Empowering Leadership, Psychological Empowerment, and Job Satisfaction directly affect Organizational Citizenship Behavior. The results of this study indicate that it is important for superiors to better understand how empowering leadership can be applied to produce a positive impact on subordinates, the superiors themselves, and the organization. Empowering Leadership can be beneficial in the relationship between subordinates and superiors and the organization as a whole [16]. The results of this study prove that applying empowering leadership impacts the officers’ psychological conditions in the form of meaningful work, confidence in competence, and ability to make decisions. In addition, the empowerment given to Police Mobile Brigade Corps officers can create a sense of satisfaction with their work. Thus, the existence of an empowering leadership style, psychological conditions for the empowerment received, and job satisfaction felt by officers can foster behavior willing to do things outside of their duties and functions voluntarily for the functional effectiveness of the Police Mobile Brigade Corps. This is essential because one of the benefits of organizational citizenship behavior is an increase in individual work productivity [53] and the ability to positively influence organizational success and performance [54].

## Limitations and suggestions

Although this study proves that Psychological Empowerment and Job Satisfaction play a role in mediating the influence between Empowering Leadership on Organizational Citizenship Behavior, this study measures variables only based on the respondent’s point of view and does not pay attention to the leader’s point of view. In addition, this research is only limited to the concept of leadership and attitudes that can drive individual behavior.

Based on the limitations that have been described, it can be recommended for further research to conduct research by measuring the variables in this model by adding the perspectives of other people, not just themselves such as the perspective of leaders or commanders in the organization, so that the research results will be much more accurate and wider. In addition, future research can also examine other variables that can encourage Organizational Citizenship Behavior, such as organizational factors, work environment, and personality. Then, = further research can also conduct research with this model on other police unit research objects.

## Supporting information

S1 File (DOCX)

S2 File (DOCX)

## References

[1] Batilmurik R, Sudiro A, Noermijati N, Rohman F. The Role Of Organizational Citizenship Behavior As Relations Mediator: Study Of Personality And Performance Of Police In Indonesia. Int J Sci Technol Res. 2020;9(6):133–40.

[2] Siregar SN. Evaluasi Sepuluh Tahun Reformasi Polri. J Penelit Polit. 2008;5(1):47–58.

[3] Organ DW. Organizational citizenship behavior: It’s construct clean-up time. Hum Perform. 1997 Jun;10(2):85–97. doi: 10.1207/s15327043hup1002_2

[4] de Geus CJC, Ingrams A, Tummers L, Pandey SK. Organizational Citizenship Behavior in the Public Sector: A Systematic Literature Review and Future Research Agenda. Public Adm Rev. 2020 Mar 14;80(2):259–70.

[5] Putra BS. The Factors Influencing Organizational Citizenship Behavior. In: Proceedings of the 5th ASEAN Conference on Psychology, Counselling, and Humanities (ACPCH 2019). Paris, France: Atlantis Press; 2020. p. 77–80. doi: 10.2991/assehr.k.200120.017

[6] Cropanzano R, Mitchell MS. Social exchange theory: An Interdisciplinary review. J Manage. 2005 Dec 1;31(6):874–900. doi: 10.1177/0149206305279602

[7] Cropanzano R, Anthony EL, Daniels SR, Hall A V. Social exchange theory: A critical review with theoretical remedies. Acad Manag Ann. 2017 Jan;11(1):479–516. doi: 10.5465/annals.2015.0099

[8] Amundsen S, Martinsen ØL. Empowering leadership: Construct clarification, conceptualization, and validation of a new scale. Leadersh Q. 2014 Jun;25(3):487–511. doi: 10.1016/j.leaqua.2013.11.009

[9] Liden RC, Wayne SJ, Sparrowe RT. An examination of the mediating role of psychological empowerment on the relations between the job, interpersonal relationships, and work outcomes. J Appl Psychol. 2000;85(3):407–16. doi: 10.1037/0021-9010.85.3.407 10900815

[10] Taylor J. Goal Setting in the Australian Public Service: Effects on Psychological Empowerment and Organizational Citizenship Behavior. Public Adm Rev. 2013 May;73(3):453–64. doi: 10.1111/puar.12040

[11] Locke EA. What is job satisfaction? Organ Behav Hum Perform. 1969 Nov 1;4(4):309–36. doi: 10.1016/0030-5073(69)90013-0

[12] Yuwono H, Gunawan DR, Eliyana A, Anggraini RD, Herlambang P, Jalil NIA. Transformational leaders’ approach to overcapacity: A study in correctional institutions. Baker R, editor. PLoS One. 2022 Nov 10;17(11 November):e0276792. doi: 10.1371/journal.pone.0276792 36356034 PMC9648837

[13] Fachry M. The implementation of impact/implication of motto dedication of brimob “my physical and spiritual life for humanity” upon the behavior changes of corps Brimob Polri personnel. Universitas Indonesia; 2010.

[14] Kaur N, Kang LS. Person-organisation fit, person-job fit and organisational citizenship behaviour: An examination of the mediating role of job satisfaction. IIMB Manag Rev. 2021 Dec;33(4):347–59. doi: 10.1016/j.iimb.2021.12.003

[15] Shahab MA, Sobari A, Udin U. Empowering leadership and organizational citizenship behavior: The mediating roles of psychological empowerment and emotional intelligence in medical service industry. Int J Econ Bus Adm. 2018;6(3):80–91.

[16] Berntzen MN, Steen FH. Empowerment and organizational citizenship behavior: An investigation of empowering leadership, psychological empowerment, organizational citizenship behaviors, and the moderating role of social and economic LMX. BI Norwegian Business School; 2013.

[17] Fong KH, Snape E. Empowering leadership, psychological empowerment and employee outcomes: Testing a multi-level mediating model. Br J Manag. 2015 Jan;26(1):126–38. doi: 10.1111/1467-8551.12048

[18] Raub S, Robert C. Differential effects of empowering leadership on in-role and extra-role employee behaviors: Exploring the role of psychological empowerment and power values. Hum Relations. 2010;63(11):1743–70.

[19] Gustari I, Widodo W. Exploring The Effect of Empowerment and GCG on OCB: Mediating by Job Satisfaction. J Xi’an Univ Archit Technol. 2020 May 9;21(5):753–61. doi: 10.37896/JXAT12.05/1473

[20] Jiang JY, Sun LY, Law KS. Job Satisfaction and Organization Structure as Moderators of the Effects of Empowerment on Organizational Citizenship Behavior: A Self-Consistency and Social Exchange Perspective. Int J Manag. 2011;28(3):675–93.

[21] Putri ER, Udin, Djastuti I. Structural Empowerment and Service-Oriented Organizational Citizenship Behavior: The Mediating Roles of Innovativeness and Job Satisfaction. Qual—Access to Success. 2019;20(170):112–7.

[22] Srivastava A, Bartol KM, Locke EA. Empowering Leadership in Management Teams: Effects on Knowledge Sharing, Efficacy, And Performance. Acad Manag J. 2006 Dec;49(6):1239–51. doi: 10.5465/amj.2006.23478718

[23] Lorinkova NM, Perry SJ. When Is Empowerment Effective? The Role of Leader-Leader Exchange in Empowering Leadership, Cynicism, and Time Theft. J Manage. 2017 May 26;43(5):1631–54. doi: 10.1177/0149206314560411

[24] Wang H, Zhang Y, Li P, Henry SE. You raise me up and I reciprocate: Linking empowering leadership to organizational citizenship behavior and unethical pro-organizational behavior. Appl Psychol. 2023 Apr 19;72(2):718–42. doi: 10.1111/apps.12398

[25] Mithulan R, Opatha HHDNP. The Moderating Effect of Personal Character and Mediating Effect of Organizational Citizenship Behavior on Ethical Orientation of HRM- Ethical Behavior Linkage. Sri Lankan J Hum Resour Manag. 2023 Jan 23;13(1):1–26. doi: 10.4038/sljhrm.v13i1.5682

[26] Bester J, Stander MW, Van Zyl LE. Leadership empowering behaviour, psychological empowerment, organisational citizenship behaviours and turnover intention in a manufacturing division. SA J Ind Psychol. 2015 Feb 5;41(1):1–14. doi: 10.4102/sajip.v41i1.1215

[27] Wong Humborstad S I., Nerstad C G.L., Dysvik A. Empowering leadership, employee goal orientations and work performance. Pers Rev. 2014 Mar 4;43(2):246–71. doi: 10.1108/PR-01-2012-0008

[28] Spreitzer GM. Psychological Empowerment in the Workplace: Dimensions, Measurement, and Validation. Acad Manag J. 1995 Oct;38(5):1442–65. doi: 10.5465/256865

[29] Lee M, Koh J. Is empowerment really a new concept? Int J Hum Resour Manag. 2001 Jan 9;12(4):684–95. doi: 10.1080/713769649

[30] Soleimani M, Dana LP, Salamzadeh A, Bouzari P, Ebrahimi P. The effect of internal branding on organisational financial performance and brand loyalty: mediating role of psychological empowerment. J Asian Bus Econ Stud. 2022 Jan 25; doi: 10.1108/JABES-08-2021-0122

[31] Kundu SC, Kumar S, Gahlawat N. Empowering leadership and job performance: mediating role of psychological empowerment. Manag Res Rev. 2019 May 20;42(5):605–24. doi: 10.1108/MRR-04-2018-0183

[32] Alotaibi SM, Amin M, Winterton J. Does emotional intelligence and empowering leadership affect psychological empowerment and work engagement? Leadersh Organ Dev J. 2020;41(8):971–91.

[33] Aksel I, Serinkan C, Kiziloglu M, Aksoy B. Assessment of Teachers’ Perceptions of Organizational Citizenship Behaviors and Psychological Empowerment: An Empirical Analysis in Turkey. Procedia—Soc Behav Sci. 2013 Oct;89:69–73. doi: 10.1016/j.sbspro.2013.08.811

[34] Li N, Chiaburu DS, Kirkman BL. Cross-Level Influences of Empowering Leadership on Citizenship Behavior. J Manage. 2017 Apr 9;43(4):1076–102. doi: 10.1177/0149206314546193

[35] Eliyana A, Sridadi AR. Workplace spirituality and job satisfaction toward job performance: The mediation role of workplace deviant behavior and workplace passion. Manag Sci Lett. 2020;10(11):2507–20. doi: 10.5267/j.msl.2020.3.044

[36] Abun D, Lucas MO, Magallanes T, Encarnation JM, Flores N. Empowering Leadership of the Heads as Perceived by the Employees and Employees’ Job satisfaction. Tech Soc Sci J. 2021;17(1):398–423.

[37] Urbini F, Chirumbolo A, Callea A. Promoting individual and organizational ocbs: The mediating role of work engagement. Behav Sci (Basel). 2020 Sep 14;10(9):138. doi: 10.3390/bs10090138 32937914 PMC7551251

[38] Yun S, Cox J, Sims H, Sabrina S. Leadership and Teamwork: The Effects of Leadership and Job Satisfaction on Team Citizenship. Int J Leadersh Stud. 2007;2(3):171–93.

[39] Sekaran U, Bougie R. Research Methods for Business: A Skill-Building Approach. 7th ed. Chichester: John Wiley & Sons, Ltd; 2016.

[40] Hair JF, Hult GTM, Ringle CM, Sarstedt M. A Primer on Partial Least Squares Structural Equation Modeling (PLS-SEM). 2nd ed. Thousand Oaks: SAGE Publications Inc.; 2017.

[41] Macdonald S, MacIntyre P. The generic job satisfaction scale: Scale development and its correlates. Empl Assist Q. 1997;13(2):1–16.

[42] Williams LJ, Anderson SE. Job Satisfaction and Organizational Commitment as Predictors of Organizational Citizenship and In-Role Behaviors. J Manage. 1991 Sep 30;17(3):601–17. doi: 10.1177/014920639101700305

[43] Henseler J, Ringle CM, Sarstedt M. A new criterion for assessing discriminant validity in variance-based structural equation modeling. J Acad Mark Sci. 2015 Jan 22;43(1):115–35. doi: 10.1007/s11747-014-0403-8

[44] Chen D, Zhang Y, Ahmad AB, Liu B. How to Fuel Public Employees’ Change-Oriented Organizational Citizenship Behavior: A Two-Wave Moderated Mediation Study. Rev Public Pers Adm. 2021 Mar 24;43(1):185–208. doi: 10.1177/0734371X211052675

[45] Zhang S, Liu Y, Li G, Zhang Z, Fa T. Chinese nurses’ innovation capacity: The influence of inclusive leadership, empowering leadership and psychological empowerment. J Nurs Manag. 2022 Sep 11;30(6):1990–9. doi: 10.1111/jonm.13654 35476276

[46] Mutonyi BR, Slåtten T, Lien G. Empowering leadership, work group cohesiveness, individual learning orientation and individual innovative behaviour in the public sector: empirical evidence from Norway. Int J Public Leadersh. 2020 May 4;16(2):175–97. doi: 10.1108/IJPL-07-2019-0045

[47] Nurimansjah RA, Ramly M, Mallongi S, Alam R. The Intervention of Job Satisfaction in Influence The Empowering Leadership and Talent Management Toward Staff Performance. J Manaj Bisnis. 2022;9(1):67–76.

[48] Shah A, Hussain N. An Empirical Study to Evaluate the Impact of Ethical Leadership on Organizational Citizenship and Innovative Behavior: Mediated by Psychological Empowerment at the Workplace. Rev Manag Sci. 2022;4(1):125–38.

[49] Tharikh SM, Ying CY, Mohamed Saad Z, Sukumaran K a/p. Managing Job Attitudes: The Roles of Job Satisfaction and Organizational Commitment on Organizational Citizenship Behaviors. Procedia Econ Financ. 2016;35(October 2015):604–11.

[50] Alkhadher O, Beehr T, Meng L. Individualism‐collectivism and nation as moderators of the job satisfaction‐organisational citizenship behaviour relationship in the United States, China, and Kuwait. Asian J Soc Psychol. 2020 Dec 19;23(4):469–82. doi: 10.1111/ajsp.12414

[51] Firdaus M, Eliyana A, Pratama AS, Cahyani A, Kamil NLM. Continuance of organizational commitment among flight attendants as an intervening variable to job performance. Probl Perspect Manag. 2022 Dec 21;20(4):507–17. doi: 10.21511/ppm.20(4).2022.38

[52] Robbins SP, Judge TA. Organizational Behavior. 18e ed. Harlow: Pearson Education Limited; 2021.

[53] Podsakoff PM, Ahearne M, MacKenzie SB. Organizational citizenship behavior and the quantity and quality of work group performance. J Appl Psychol. 1997;82(2):262–70. doi: 10.1037/0021-9010.82.2.262 9109284

[54] Mohd. Kumar Muzamil, Shah Shawkat Ahmad. Psychometric Properties of Podsakoff’s Organizational Citizenship Behaviour Scale in the Asian Context. Int J Indian Psychol. 2015 Dec 25;3(1). doi: 10.25215/0301.152

