# Supplementary material for: Examining antecedents of organizational citizenship behavior: An empirical study in Indonesian police context
Source: PLoS One. 2023 Oct 5;18(10):e0291815. doi: 10.1371/journal.pone.0291815 (PMC10553346; doi:10.1371/journal.pone.0291815)
Supplement: S2 File — (DOCX) [file pone.0291815.s002.docx]

**Herewith the English translation for the Ethical Approval documentation:**

To Prof. Dr. Anis Eliyana, S.E., M.Sc.,

We have received your application for ethical approval and after studying the details of the research, we have decided that the research protocol entitled "Building Organizational Citizenship Behavior in the Police Context: An Empirical Study in Indonesia" during the research period June 2022 - October 2022, does not require ethical approval of two consideration:

1. The research does not involve a vulnerable population

2. The research esearch does not involve specific treatment of respondents

Greetings,

Rumayya, SE., M.Reg.Dev., Ph.D.

Head of Center for Research and Publications

Faculty of Economics and Business,

Airlangga University
