# Supplementary material for: Examining antecedents of organizational citizenship behavior: An empirical study in Indonesian police context
Source: PLoS One. 2023 Oct 5;18(10):e0291815. doi: 10.1371/journal.pone.0291815 (PMC10553346; doi:10.1371/journal.pone.0291815)
Supplement: Supplementary file 2 [file pone.0291815.s001.docx]

PARTICIPATION CONSENT

Respected Mr./Ms. Respondent,

We appreciate your willingness to access this questionnaire. In the framework of research performed by Airlangga University, you will be asked to complete a questionnaire. This questionnaire requests personal data (gender, age, marital status, tenure, and most recent education) and research variable questions (Empowering Leadership, Psychological Empowerment, Job Satisfaction, dan Organizational Citizenship Behavior).

Your responses to this questionnaire will be used solely for data analysis in this study, which will be published in international publications. The results of the research will be interpreted in a manner that ensures your anonymity. Researchers utilize and publish only anonymized data. The respondents have the right to view the outcomes of the data interpretation once the research has been finished.

Your participation in this research is entirely voluntary. This indicates that you have the option to continue filling out this form. If you opt to withdraw during the application process, there will be no consequences. By checking the box below, you'll be redirected to the questionnaire page.

I consent to participate in this study by completing this questionnaire in its entirety. I now declare, without pressure, that the answer I typed is the correct one.

- [x] Agree

Kembali | Berikutnya | Kosongkan formulir
